# Strength Enhancement of 3D-Printed Phosphogypsum Concrete Based on Synergistic Activation of Multi-Solid Wastes

**DOI:** 10.3390/ma19030482

**Published:** 2026-01-25

**Authors:** Junjie Li, Yangbo Li, Xianqiang Ge, Ke Li, Yahui Yang, Shuo Wang

**Affiliations:** 1 College of Hydraulic and Environmental Engineering, China Three Gorges University, Yichang 443002, China; 18296739885@163.com (J.L.); liyangbo@ctgu.edu.cn (Y.L.); 202308010021003@ctgu.edu.cn (S.W.); 2 POWERCHINA BEIJING Engineering Corporation Limited, Beijing 100024, China; gexianqiang@bjy.powerchina.cn (X.G.); lik@bjy.powerchina.cn (K.L.)

**Keywords:** 3D-printed concrete, phosphogypsum, strength enhancement, printability, synergistic activation, multi-solid wastes, cement replacement ratio, blast-furnace slag

## Abstract

**Highlights:**

**What are the main findings?**

- Proposes a novel route for large-scale phosphogypsum valorization in 3D-printed concrete.
- Achieves dual-function utilization of phosphogypsum as a binder and sulfate activator.
- Elucidates the synergistic mechanism between slag and phosphogypsum.

**What are the implications of the main findings?**

- Resolves the inherent strength-deficiency of 3DPPGC.
- Integrates solid waste recycling with low-carbon construction goals.
- Paves the way for large-scale engineering deployment of phosphogypsum-based materials.

**Abstract:**

Phosphogypsum (PG) is the main by-product of wet-process phosphoric acid production. Its annual global production reaches about 200 million tons, yet its utilization rate remains low. Consequently, long-term stockpiling of large PG volumes poses immense pressure to the ecological environment. To mitigate negative environmental impacts, the utilization of PG is imperative. Despite progress in PG utilization and 3D-printing technology, there is still a significant lack of understanding about the synergistic activation mechanisms in multi-solid-waste systems. In particular, the composition design, microstructure evolution, and structure–property relationships of 3D-printed PG-based composites are not well-studied, which limits their high-value engineering applications. Three-dimensional-printed phosphogypsum concrete (3DPPGC) is proposed here, promoting PG resource utilization by leveraging the expanding applications of 3D-printed concrete (3DPC). However, the strength of 3DPPGC needs to be enhanced to meet engineering requirements. This study designed the mix proportion of 3DPPGC and fabricated the corresponding test specimens. The optimal Cement Replacement Ratio (CRR) was determined through experimental testing, and the mechanism behind the strength enhancement of the 3DPPGC was elucidated. The results indicated that the 3DPPGC’s mechanical properties peaked at the 70% CRR. Compared with cast specimens, 3DPPGC exhibited a 1.52% increase in 28-day flexural strength in the y-direction, reaching 4.69 MPa. The early-age compressive strength, flexural strength, and later-age compressive strength of 3DPPGC were significantly enhanced when PG, blast-furnace slag (BS), fly ash (FA), and silica fume (SF) were used to partially replace cement. This study provides a theoretical and experimental basis for the large-scale, high-value application of PG in intelligent construction.

## 1. Introduction

With the sustainable development of the global construction industry, 3D-printed concrete (3DPC) has emerged as an innovative construction technology that is promoting the revolution of the building industry due to its advantages, such as design flexibility, formwork-free construction, rapid construction speed, and high material-utilization efficiency [1,2,3,4,5,6]—capable of reducing construction waste by 30–60%, shortening project timelines by 50–70%, and lowering labor costs by 50–80% [7,8]. Because of these significant advantages, 3DPC now has broader applications in fields ranging from residential buildings, municipal facilities, and landscape projects to special structures and emergency construction.

**Figure 1:**
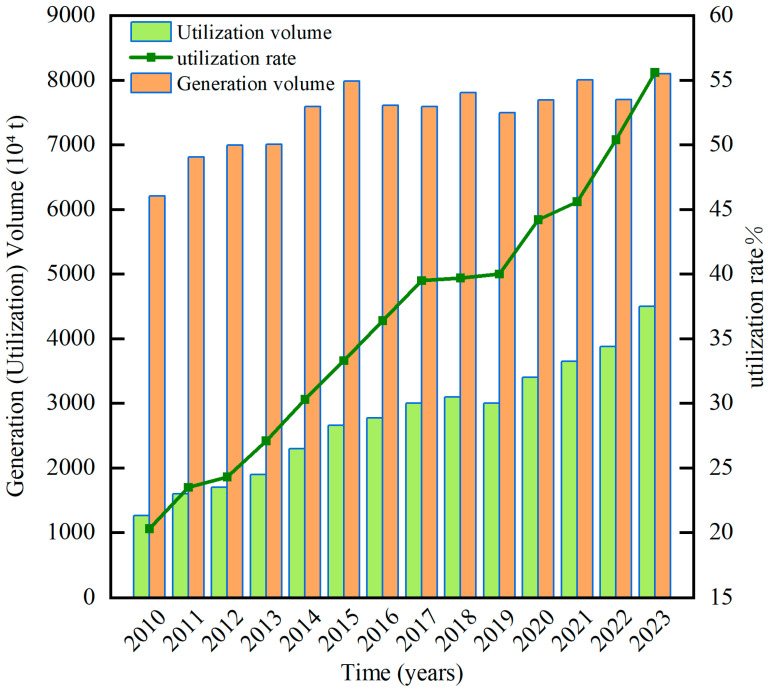
Annual comprehensive utilization rate of PG in China from 2010 to 2023.

Concurrently, phosphogypsum (PG) is the principal by-product of the wet-process phosphoric acid industry, and it poses a formidable environmental challenge. Open-air stockpiling not only monopolizes scarce land resources but also releases deleterious constituents, such as phosphorus, fluorine, heavy metals, and radionuclides. These pollutants contaminate the soil, groundwater, and atmosphere, thereby jeopardizing human health [9,10,11,12,13,14]. Globally, the cumulative inventory of PG has reached over 6 × 10^9^ t, with an annual accretion of approximately 2 × 10^8^ t; yet, the overall utilization rate remains merely ~10% [15]. In China, the stockpile has reached 4 × 10^8^ t [16], although the comprehensive utilization rate has increased year-on-year [17], it continues to lag far behind the incremental accumulation, thereby intensifying the environmental burden. Figure 1 depicts the temporal evolution of PG comprehensive utilization in China.

In the future, as 3DPC is used more widely in construction, using PG as a raw material for 3DPC can help us consume large amounts of it. This approach will support the Chinese dual carbon goals and offers great economic and environmental benefits [18,19]. Current PG valorization research predominantly focuses on its modification for gypsum blocks, wall panels, self-leveling scaffolds, subgrade materials, and cement set-retarders [20,21,22]. Properly processed PG is a high-quality cementitious material source. Its primary component, calcium sulfate dihydrate, provides good mechanical properties and promising application prospects [21].

Meanwhile, research on 3DPC technology has concentrated on modulating the printability, extrudability, buildability, and early-age strength of concrete materials [23,24]. Substantial advances in the fresh-state workability of the mixes have been achieved by optimizing the binder system, incorporating supplementary cementitious materials, introducing chemical admixtures, and adding fibers [23,24,25,26,27,28].

Although the resource utilization of PG in 3DPC shows great potential, there is still a noticeable scientific gap in current research in this field. The 3D-printing process requires materials with thixotropy and rapid early-strength development to support continuous stacking. However, soluble impurities such as phosphorus and fluorine remaining in PG can adsorb onto the surface of cement particles and form insoluble films, severely hindering ion diffusion. This leads to an extended hydration induction period and slow early-strength development [18,22,29,30,31]. The inherent slow-setting property of this material significantly conflicts with the fast construction requirements of 3D printing, and this contradiction has not been effectively resolved yet. Current research mainly focuses on preparing 3DPPGC materials and evaluating their printability. However, studies on the mechanical performance after hardening, especially the long-term strength development and anisotropic behavior, are still insufficient and lack systematic investigation [22,32]. Secondly, while the synergistic effect between PG and supplementary cementitious materials like blast-furnace slag (BS) is acknowledged [33,34], the synergistic activation mechanism within a multi-solid-waste 3DPC system lacks in-depth explanation. Key scientific issues remain unclear, including the activation pathway of sulfates on slag hydration and their quantitative impact on the sequence of hydration products. Additionally, the hardened strength of existing 3D-printed PG-based materials, particularly the flexural strength, is generally low and insufficient to meet structural-engineering standards [32]. Currently, methods for enhancing the mechanical properties of 3DPPGC are largely adapted from conventional concrete technology. However, these approaches often fail to fully account for the complex interplay between the intrinsic characteristics of the PG matrix and the 3D-printing process. How to use chemical activation to overcome impurity interference, achieve densification of the microstructure, and realize a breakthrough in mechanical performance constitutes a critical scientific gap that urgently needs to be filled in this field. Therefore, systematically investigating the influence of cement replacement rates on the strength enhancement of 3DPPGC and elucidating the underlying synergistic enhancement mechanisms are crucial for filling the aforementioned scientific gap and promoting the large-scale, high-value utilization of PG in intelligent construction. To address the issue of low strength in PGC, Sahmenko et al. [35]. pioneered the development of a gypsum–cement–pozzolan (GCP) ternary composite for 3D printing. The GCP mixture enabled continuous printing of 35 layers with good stability. Its 28-day compressive strength reached 37 MPa, comparable to conventional cement-based printing materials. Sinka et al. [32] converted dihydrate PG into cementitious hemihydrate PG via calcination. They added quicklime to neutralize acidic impurities. They successfully prepared a printable PG mortar mixture by adjusting the ratio of retarder and water reducer. The study found that its mechanical properties surpassed those of cast specimens. The compressive strength along the printing direction reached up to 950 kPa. This value was 17% higher than that of cast samples. Liu et al. [36]. significantly improved the early-age properties of PG-based cementitious materials by adding sodium aluminate. Experimental results showed that 1% sodium aluminate increased the 3-day compressive strength by 587.39% and shortened the final setting time by over 4 h. Tarhan et al. [37]. used PG and borogypsum as partial cement replacements in 3DPC. Their study found that in the ground granulated BS system, PG additions of 5%~7.5% significantly enhanced compressive strength. The 28-day strength reached 51 MPa, outperforming the control group.

At present, research on the strength enhancement of 3DPC is predominantly oriented along five axes: (1) Optimizing the mix design by refining particle packing and the interfacial transition zone through tailored proportioning, thereby enhancing matrix density and compressive strength [21]. (2) Modulating rheological properties and post-hardening mechanical performance via water-reducing agents, early-strength admixtures, or other functional additives [18,29,38]. (3) Introducing short discrete fibers to augment tensile and flexural capacity while mitigating crack propagation [20,22]. (4)Tuning print paths, nozzle geometry, and inter-layer bonding strategies to improve structural load-bearing behavior [22]. (5) Embedding steel bars or wire meshes to enhance flexural and tensile strength is an approach that is presently at the exploratory stage [39].

Moreover, in terms of material proportioning and hydration mechanisms, Huang and Lin [40] provided experimental data and a theoretical foundation for the mix design of this study, the analysis of strength enhancement mechanisms, and the feasibility of engineering applications. Their work particularly formed a critical support in elucidating the synergistic activation effects between PG and slag.

Zhang et al. [34] prepared PG-based composite cementitious materials using PG, cement, slag powder, fly ash (FA), retarders, and water reducers. They found that the hydration products transformed from needle-like to rod-like or plate-like structures. These products were adhered to the structural framework by extensive reticular and filamentous C-S-H gels, resulting in a dense matrix. These findings provide a theoretical foundation and critical support for the strength enhancement mechanism of the 3D-printed PGC in this study.

To address the key scientific challenges of insufficient hardened strength in 3D-printed phosphogypsum concrete (3DPPGC) and the unclear synergistic activation mechanism in multi-solid-waste systems, this study aims to enhance the strength of 3DPPGC by partially replacing cement with PG, blast furnace slag (BS), and FA. The main objectives are to investigate the influence of the Cement Replacement Ratio (CRR) on the mechanical properties and to determine an optimal mix proportion that balances excellent printability with high strength. The specific tasks include: (1) systematically designing mixtures with different CRRs and evaluating their fresh-state printing performance (including fluidity, extrudability, and buildability); (2) studying the evolution of flexural and compressive strength at curing ages of 3, 7, 14, and 28 days and analyzing the mechanical anisotropy induced by the printing process; (3) elucidating the underlying synergistic strengthening mechanism through which PG induces a sulfate activation effect in the slag–cement system. Ultimately, this research aims to develop a high-performance 3DPPGC, providing a solid theoretical and experimental basis for the large-scale, high-value application of PG in intelligent construction, thereby promoting green and sustainable development in the building industry.

## 2. Experimental Raw Materials and Protocol Design

### 2.1. Experimental Raw Materials

**Figure 2:**
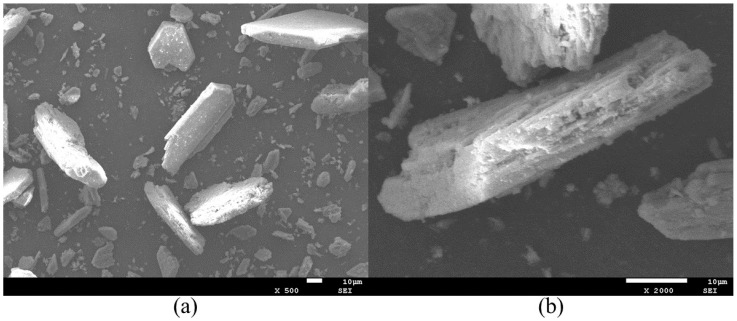
SEM images of PG: (**a**) magnification 500×; (**b**) magnification 2000×.

The PG utilized in this study was sourced as a gray, powdery by-product from a phosphorus chemical plant in Yichang City, Hubei Province, China. Its physical properties include a bulk density of 1950 kg/m^3^ and a moisture content of 16%. According to standard measurement [41], the pH value of the as-received material was 4. Prior to testing, the raw PG was oven-dried at 60 °C and subsequently characterized by the Blaine method, yielding a specific surface area of 192 m^2^/kg. X-ray Diffraction (XRD) analysis revealed that the primary mineralogical constituents are CaSO_4_·2H_2_O and SiO_2_, confirming that PG serves as the principal source of sulfate and siliceous phases within phosphogypsum concrete (PGC). The SEM image of PG is shown in Figure 2.

**Figure 3:**
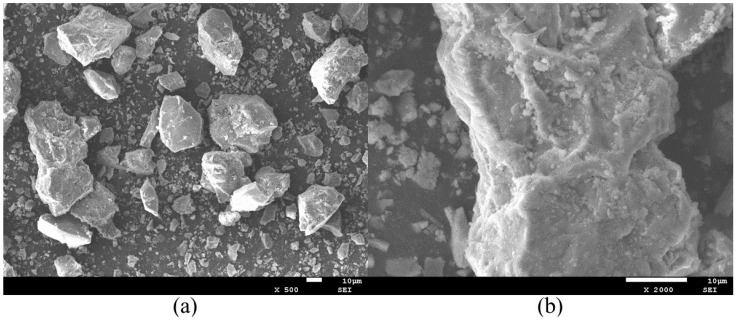
SEM images of cement: (**a**) magnified 500×; (**b**) magnified 2000×.

The Ordinary Portland Cement employed was Huaxin P·O 42.5 reference cement, sourced from Wuhan, Hubei Province, China, exhibiting a bulk density of 1.30 × 10^3^ kg/m^3^ and a specific surface area of 381.7 m^2^/kg. The SEM image of Ordinary Portland Cement is shown in Figure 3.

**Figure 4:**
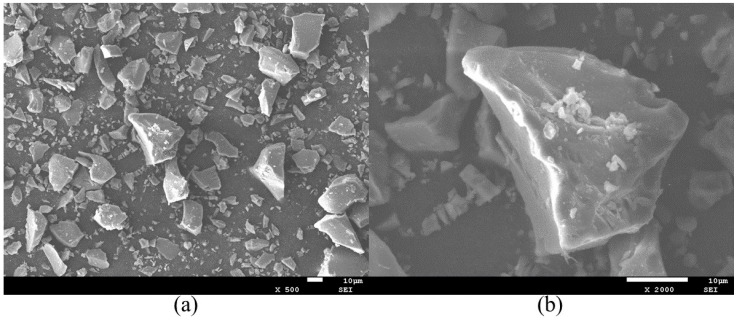
SEM images of BS: (**a**) magnified 500×; (**b**) magnified 2000×.

BS, sourced from Gongyi Longze Water-Purifying Materials Co., Ltd. (Gongyi, China), is an off-white, solid powder exhibiting acicular or platy morphologies. Its tapped bulk density is 1250 kg/m^3^, and its specific surface area is 429 m^2^/kg. The SEM image of BS is shown in Figure 4.

**Figure 5:**
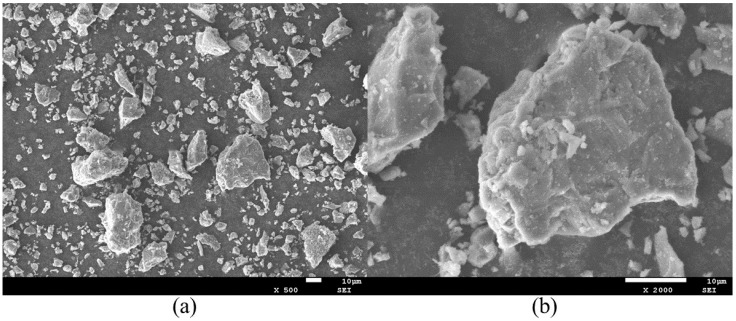
SEM images of FA: (**a**) magnified 500×; (**b**) magnified 2000×.

FA was procured from a coal-fired power plant in Wuhan City, China and appears as a gray, solid powder. Its loose bulk density is 2440 kg/m^3^ and its specific surface area is 962 m^2^/kg. The SEM image of FA is shown in Figure 5.

**Figure 6:**
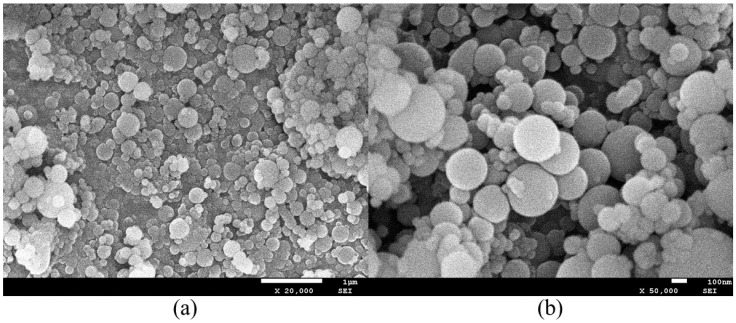
SEM images of SF: (**a**) magnified 20,000×; (**b**) magnified 50,000×.

SF, the predominant amorphous form of SiO_2_, consists of over 90% spherical SiO_2_ particles by mass. These particles are extremely fine, with an average diameter ranging from 0.1 to 0.3 μm. Its specific gravity is 2.35 × 10^3^ kg/m^3^, the bulk density is 1.26 × 10^3^ kg/m^3^, and the specific surface area is 2.34 × 10^3^ m^2^/kg. The chemical compositions of all constituent materials are presented in Table 1. The SEM image of SF is shown in Figure 6.

In this study, natural sand with a particle-size distribution confined to the range of 0.5–1 mm served as the fine aggregate.

Polycarboxylate-based high-range water-reducing admixture (WR): supplied by Shanghai Chenqi Chemical Technology Co., Ltd. (Shanghai, China); pH = 7; water-reduction rate of 19% in mortar and 32% in concrete.

Triethanolamine (TEOA): supplied by Tianjin Zhonglian Chemical Reagents Co., Ltd. (Tianjin, China); chloride content 0.0009%, sulfate content 0.0008%. TEOA not only improves the waterproofing and impermeability of the material but also enhances the early-age strength of concrete [42].

CaO: employed to neutralize acidic impurities in PG, converting soluble phosphorus and fluorine into insoluble Ca_3_(PO_4_)_2_ and CaF_2_, thereby mitigating the deleterious effects of P and F on the performance of PGC.

### 2.2. Mixture Proportion Design

The present investigation identifies 20 wt% PG as the optimal replacement level, which markedly enhances the mechanical performance of the composite cementitious system [40]. Four mix proportions of PGC were designed. The cement content was progressively reduced from 50% to 20% (by mass), while the combined dosage of BS, FA, and SF was increased from 30% to 60%. A binder-to-sand ratio of 1:1 and a water-to-binder ratio of 0.40 were adopted throughout. The FA and SF were incorporated at fixed levels of 14% and 7% of the cement mass, respectively, to prevent deleterious strength losses associated with excessive supplementary cementitious materials [43]. PGC-0 served as the control specimen. A 2.5 m class concrete 3D printer was employed, configured with a layer thickness of 10 mm, a print-head travel speed of 3 cm/s, an extrusion rate of 5 cm/s, and a nozzle diameter of 20 mm. The binder contents are listed in Table 2, and the mix proportions of 3DPPGC are provided in Table 3, where CRR denotes the combined replacement ratio of cement by PG, BS, FA, and SF.

Prior to batching, quicklime and oven-dried PG were blended in accordance with the proportions specified in Table 3. and aged for 24 h. The mixture was subsequently subjected to 2 min of manual stirring, followed by 2 min of low-speed mechanical agitation and 3 min of high-speed mixing to ensure complete homogenization of all constituents. To fully eliminate entrapped air, the container was vibrated on a vibrating table for 1 min.

### 2.3. Experimental Setup

All mixing, printing, and subsequent curing of the specimens were conducted in a controlled laboratory environment. The ambient temperature was maintained at 23 ± 2 °C, and the relative humidity was 60 ± 5%. These conditions were monitored throughout the experimental process to ensure consistency.

The extrusion process was controlled by maintaining a constant extrusion rate and print-head travel speed. Table 4 presents the specific printing parameters.

The 2.5 m class concrete 3D printer was calibrated prior to the printing tests. The positional accuracy of the print-head in the X, Y, and Z axes was within ±0.1 mm, and the repeatability was within ±0.05 mm. This calibration ensured the dimensional accuracy and consistency of the printed specimens.

## 3. Experimental Procedures

### 3.1. Setting-Time and Flowability Tests

The setting-time test was conducted in accordance with the standard [44] by means of a Vicat apparatus, while the fluidity test was performed on an NLD-3 cement mortar flow-table following the standard [45].

### 3.2. Extrudability Assessment

**Figure 7:**
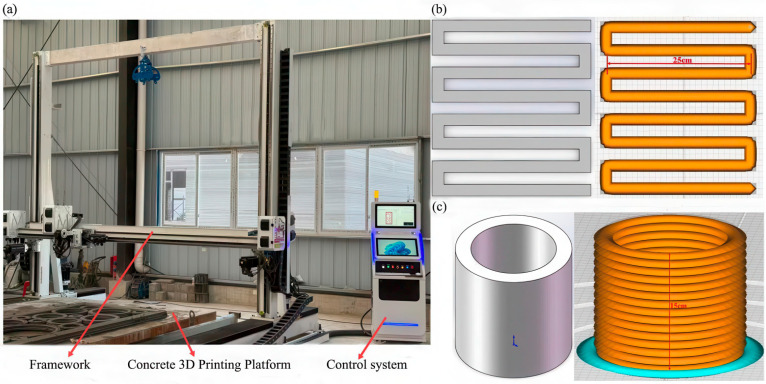
(**a**) Concrete 3D printer. (**b**) Three-dimensional model and slicing pattern of the extrudability test specimen. (**c**) Buildability model and its slicing representation.

Following the methodology proposed by Lafhaj et al. [46], the continuity of the printed filaments depicted in Figure 7b was visually examined, and the extruded strand width was precisely measured. According to the extrudability evaluation index expressed in Equation (1), a value of *J_c_* slightly greater than unity is typically adopted to ensure adequate structural stability of the printed construct.

(1) $$J_{c}=\frac{d}{D}\times 100\%$$

In Equation (1), *J_c_* denotes the extrusion ratio, *d* represents the actual extruded width of the concrete filament, and *D* corresponds to the internal diameter of the printing nozzle.

### 3.3. Buildability Evaluation

Following the method proposed by Bhattacherjee et al. [47], a hollow cylinder with an outer diameter of 150 mm, an inner diameter of 130 mm, and a height of 150 mm (illustrated in Figure 7c) was fabricated in a single continuous print. Immediately upon completion of printing, the initial height was measured, and subsequently re-measured at 10 min intervals to record the overall settlement and to compute the *J_z_* value. To enable a more rigorous quantification of buildability, Equation (2) was employed:

(2) $$J_{z}=\frac{h}{D_{n}}\times 100\%$$

where *J_z_* is the build ratio, *h* is the actual height of the specimen, *n* is the total number of printed layers, and *D_n_* is the theoretical height of the n-th layer. Provided that the printer accuracy is sufficient, *J_z_* is generally slightly below one owing to the cumulative pressure of overlying layers and the self-compaction of the extruded concrete filaments.

### 3.4. Mechanical Property Characterization

**Figure 8:**
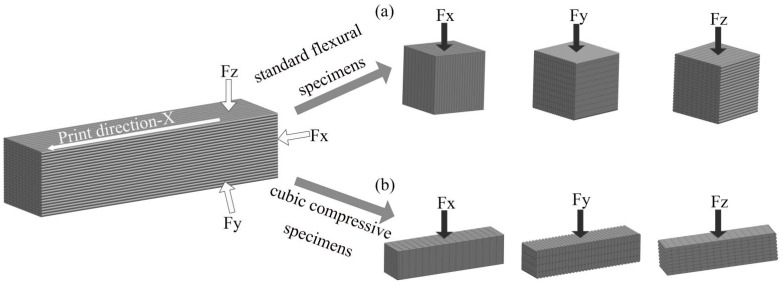
(**a**) Schematic illustration of the different loading directions applied to standard flexural specimens. (**b**) Schematic illustration of the different loading directions applied to cubic compressive specimens.

A total of 288 specimens were fabricated using 3D-printing technology based on the mix proportions specified in Table 3, comprising 144 prism specimens (40 mm × 40 mm × 160 mm) for flexural strength testing [48] and 144 cubic specimens (100 mm) for compressive strength evaluation. The mechanical properties of the cured specimens were determined at curing ages of 3, 7, 14, and 28 days in compliance with the Chinese National Standard [49], with separate measurements conducted for flexural and compressive strengths. The distinct loading orientations for flexural and compressive specimens are illustrated in Figure 8.

The cast specimens were prepared using the same batch of PGC slurry, with identical raw materials and mix proportions as the 3D-printed specimens. The mixture was compacted using a vibrating table. The fresh mixture was filled in a single layer into standard molds (flexural test: 40 mm × 40 mm × 160 mm prisms; compressive test: 100 mm cubes) conforming to the Chinese National Standard [49]. During filling, a trowel was used to poke along the inner walls of the molds to remove air pockets. The molds were fixed onto the vibrating table and vibrated until the surface became level and cement paste appeared, avoiding over-vibration. After molding, excess mixture was scraped off, and the surface was smoothed with a trowel just before the concrete reached its initial setting. All cast specimens were covered with waterproof plastic-film immediately after molding to prevent moisture evaporation. They were kept at room temperature (20 ± 5 °C) for 24 h before demolding. Subsequently, the cast specimens were placed in a standard curing room (temperature 20 ± 2 °C, relative humidity > 95%) together with the 3D-printed specimens. The specimens were spaced apart to ensure all surfaces were freely exposed to the moist air.

**Figure 9:**
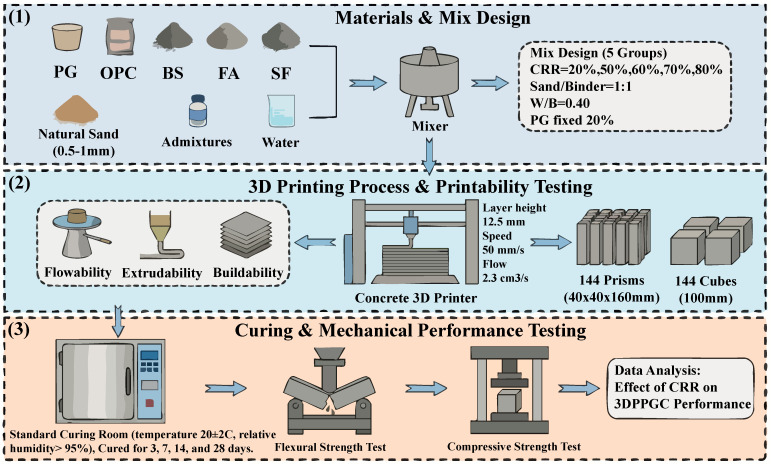
Flowchart for the preparation and performance testing of 3DPPGC. (1) Materials & Mix Designl; (2) 3D Printing Process & Printability Testing; (3) Curing & Mechanical Performance Testing.

All mechanical property tests were conducted on at least three replicate specimens, and the results are reported as mean ± standard deviation (SD). The standard deviation was used to assess the data dispersion and is presented as error bars in the figures. Figure 9 illustrates the specific experimental process.

## 4. Results and Discussion

### 4.1. Setting Time

**Figure 10:**
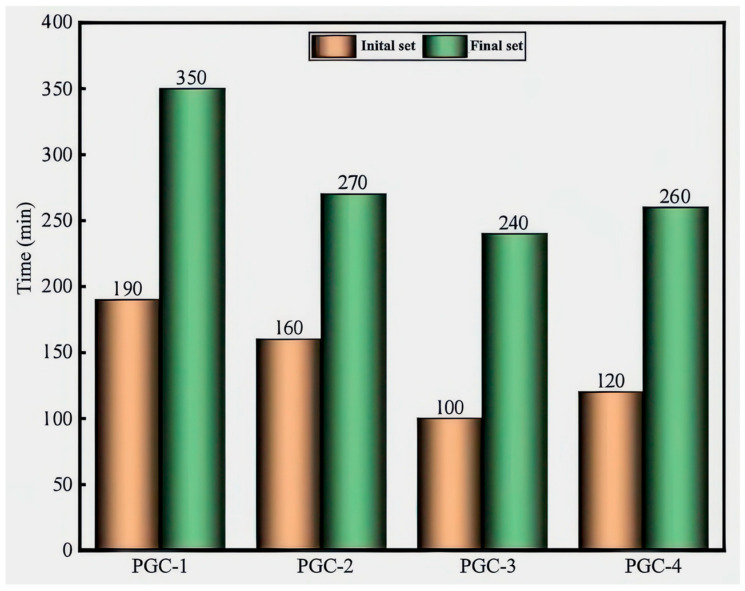
Setting-time results of PGCs with varying CRRs.

As illustrated in Figure 10, the incorporation of BS markedly accelerates the hydration reaction and effectively shortens the setting time. With increasing CRR, the setting time of PGC initially decreases and then increases, reaching its minimum at a CRR of 70%. Previous investigations [50,51] have demonstrated that the sulfate ions released from gypsum can elicit a sulfate-activation effect; concurrently, the substantial quantity of OH^−^ generated by the hydration of pre-blended quicklime and PG synergistically activates the BS in the presence of sulfates, thereby accelerating the overall hydration kinetics [40,52,53]. It is noteworthy that OH^−^ within PGC is also supplied by the hydration of Portland cement. When the combined content of PG and cement approximates the dosage of BS, the hydration rate reaches its maximum and the setting time is minimized. Conversely, when the combined content of PG and cement is lower than that of BS, the hydration rate declines, resulting in prolonged setting time. Therefore, the rational proportioning of cement, PG, BS, FA, and SF is essential for regulating the setting behavior of PGC, and the judicious incorporation of chemical activators such as SO_4_^2−^ can further enhance the efficiency of the hydration reactions.

### 4.2. Flowability

**Figure 11:**
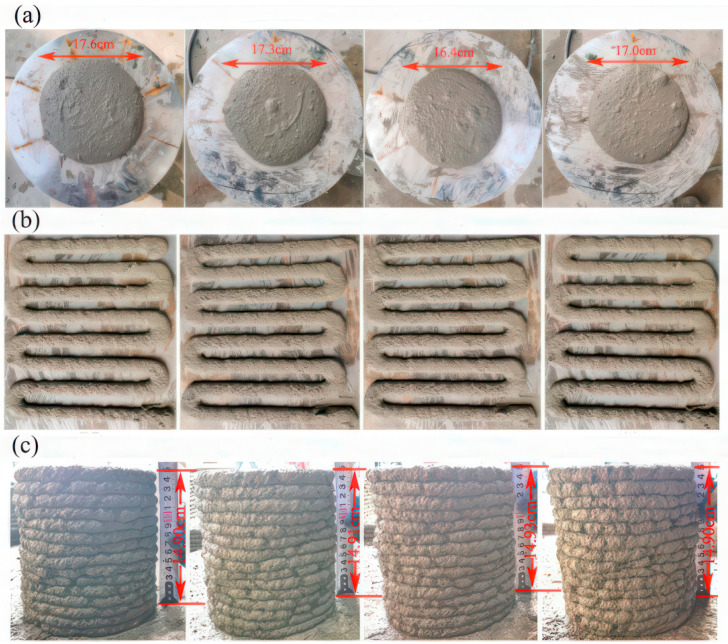
(**a**) Flow-table test results at 0 min for PGC with varying CRRs. (**b**) Extrudability test results of PGC with various CRR contents. (**c**) Buildability test results of PGC with various CRR contents. (From the left, CRR is 50%, 60%, 70%, and 80%, respectively).

Figure 11a illustrates that, at 0 min, the flow-table tests of groups PGC-1 to PGC-4 exhibited approximately circular and uniformly distributed spreads, indicating that the fresh PGC possesses excellent initial fluidity, which is conducive to pumping and extrusion in 3D printing.

**Figure 12:**
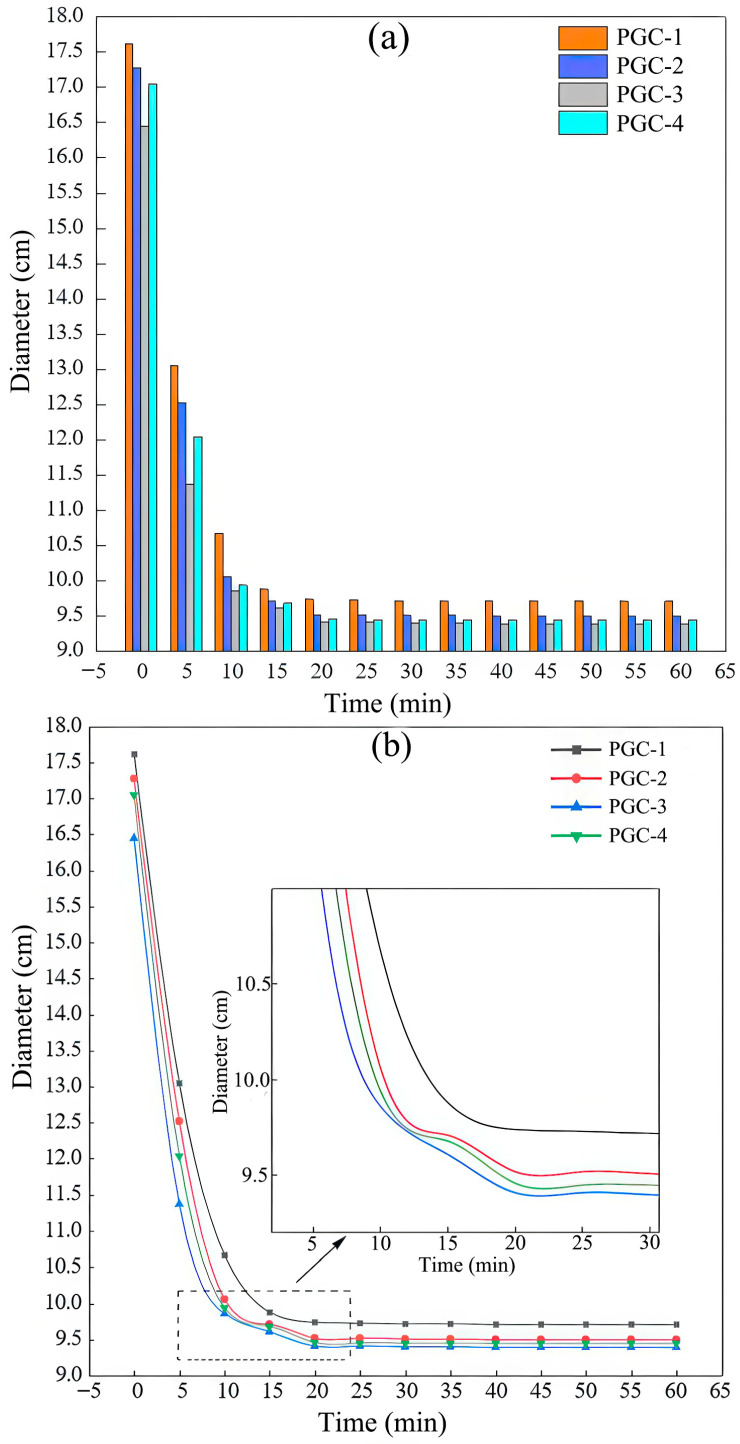
Flow-table test results of PGC with various CRR contents within 60 min. (**a**) Histogram. (**b**) Curve plot and local magnification view.

During the flow-table test, samples were taken every 5 min, and the resulting diameters measured within 60 min were plotted as both a bar chart and a curve, as shown in Figure 12a,b.

Figure 12a presents the initial spread diameters of PGC-1 through PGC-4, which are 17.6 cm, 17.3 cm, 16.4 cm, and 17.0 cm, respectively. According to Figure 12b, the flow diameters of all mixtures decrease sharply within the first 10 min and then stabilize at 9.7 cm, 9.5 cm, 9.4 cm, and 9.4 cm after 20 min. The corresponding reductions amount to 44.89%, 45.09%, 42.68%, and 44.71%, respectively. The rate of reduction first decreases and subsequently increases, which can be attributed to the highest hydration rate observed in the PGC-3 mixture; this accelerated hydration also accounts for its smaller initial diameter. Notably, PGC-3 exhibits the smallest flow diameter at every measurement interval, again reflecting its elevated hydration kinetics. This observation aligns with the setting-time data. The hydration rate is maximized when the combined dosage of PG and cement is approximately equal to that of BS, and the overall hydration rate of the system is markedly accelerated.

### 4.3. Extrudability

As illustrated in Figure 11b, the average strip widths of the PGC-1 to PGC-4 mixtures were measured as 2.01 cm, 2.01 cm, 2.01 cm, and 1.96 cm, respectively. These values are all extremely close to the target width of 2 cm, with a maximum deviation of merely 0.04 cm, which is negligible. The corresponding *J_c_* values for PGC-1 to PGC-4 were 100.50%, 100.50%, 100.50%, and 98.00%, respectively. These results indicate that all four mix designs exhibit excellent extrudability.

### 4.4. Buildability

The buildability test results are depicted in Figure 11c. The experiments reveal that the material’s pronounced viscosity induces pronounced adhesion to the inner wall of the nozzle, resulting in an extruded filament diameter slightly below 2 cm and the formation of micro-cracks along its outer periphery.

**Figure 13:**
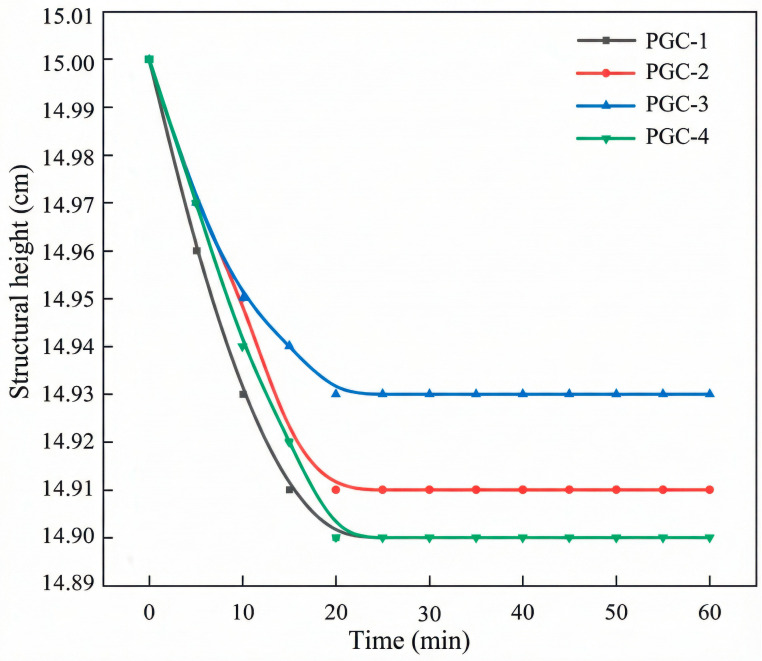
Buildability test results of PGC with various CRR contents within 60 min.

As illustrated in Figure 13, the initial heights of specimens PGC-1 through PGC-4 were uniformly 15.00 cm, whereas their final structural heights were measured to be 14.90 cm, 14.91 cm, 14.93 cm, and 14.90 cm, respectively. The corresponding *J_z_* values are 99.33%, 99.4%, 99.53%, and 99.33%.

The heights of all specimens stabilized after 20 min, which is consistent with the results obtained from the slump-flow test. This indicates that the material’s yield stress had become sufficient to support its own weight, thereby confirming the excellent buildability of each PGC mixture. After 20 min, the heights of all specimens stabilized, in full agreement with the flowability results, indicating that the yield stress of the material had become sufficient to sustain its own weight; this confirms that every PGC mixture possesses excellent buildability.

### 4.5. Mechanical Performance: Experimental Results and Analysis

#### 4.5.1. Flexural Strength

**Figure 14:**
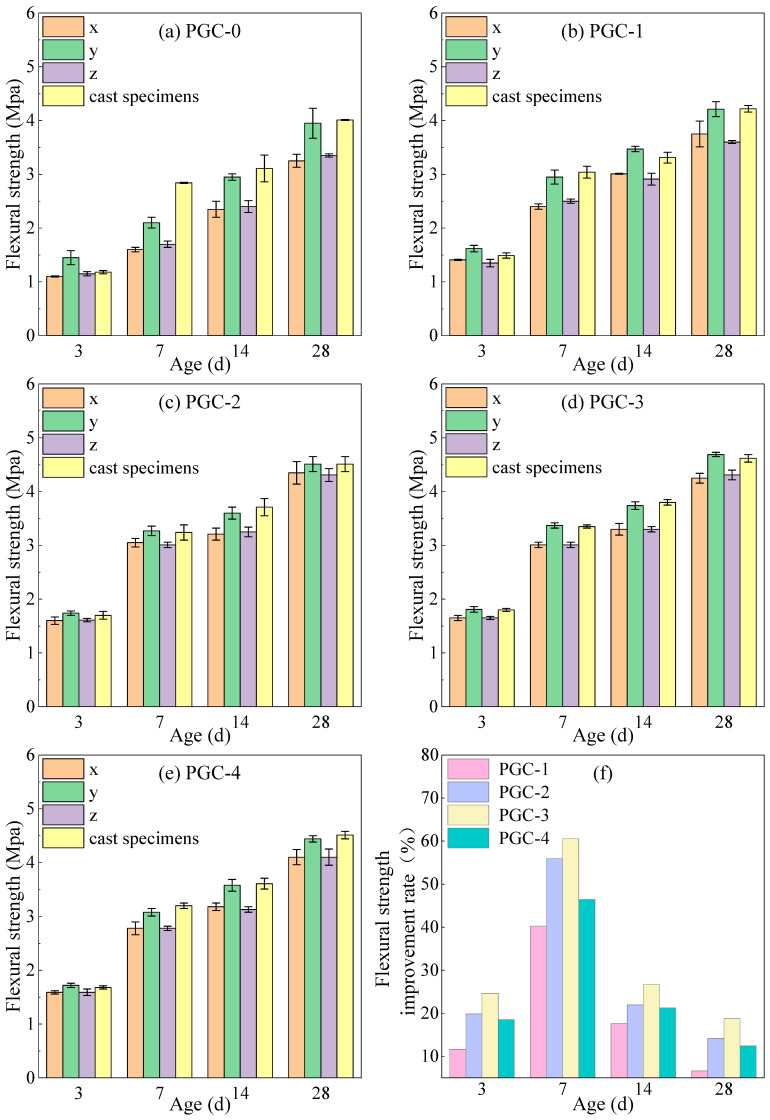
Flexural strength test results (mean ± SD, n = 3). (**a**) Control group. (**b**) CRR is 50%. (**c**) CRR is 60%. (**d**) CRR is 70%. (**e**) CRR is 80%. (**f**) Flexural strength increase (%) over PGC-0.

The flexural strength test results for the PGC-1–PGC-4 and PGC-0 specimens at curing ages of 3 d, 7 d, 14 d, and 28 d are presented in Figure 14.

According to Figure 14a–e, following partial cement replacement with BS, FA, and SF, the flexural strength of 3DPPGC exhibited a consistent enhancement across all curing ages. The flexural strength first increased and then decreased with rising CRR. When CRR reached 70%, the flexural strength of group PGC-3 in the y-direction with the 28-day age attained 4.69 MPa, equivalent to 101.52% of that of the conventionally cast counterpart; the remaining groups registered 91.13%, 97.62%, and 96.10%, respectively. These results demonstrate that the flexural performance of 3D-printed specimens fulfills the requirements for engineering applications. As shown in Figure 14f, all groups from PGC-1 to PGC-4 had higher flexural strength in the y-direction than control group PGC-0. PGC-3 showed the best improvement. At 3, 7, 14, and 28 days, its strength increased by 24.66%, 60.56%, 26.69%, and 18.73%. All groups reached their highest increase at 7 days. The increase then fell at 14 and 28 days. However, PGC-3 still had a high improvement rate and performed the best.

As presented in Table 5, the flexural-strength growth rate of 3DPPGC specimens along the y-direction during the 0–7 d age interval closely matches that of cast specimens, yet exceeds that of the PGC-0 group, demonstrating that the incorporation of BS, FA, and SF markedly accelerates early-age strength development of the PG-based matrix. This growth rate increases monotonically with CRR, attaining its maximum at a CRR of 70%.

#### 4.5.2. Compressive Strength

**Figure 15:**
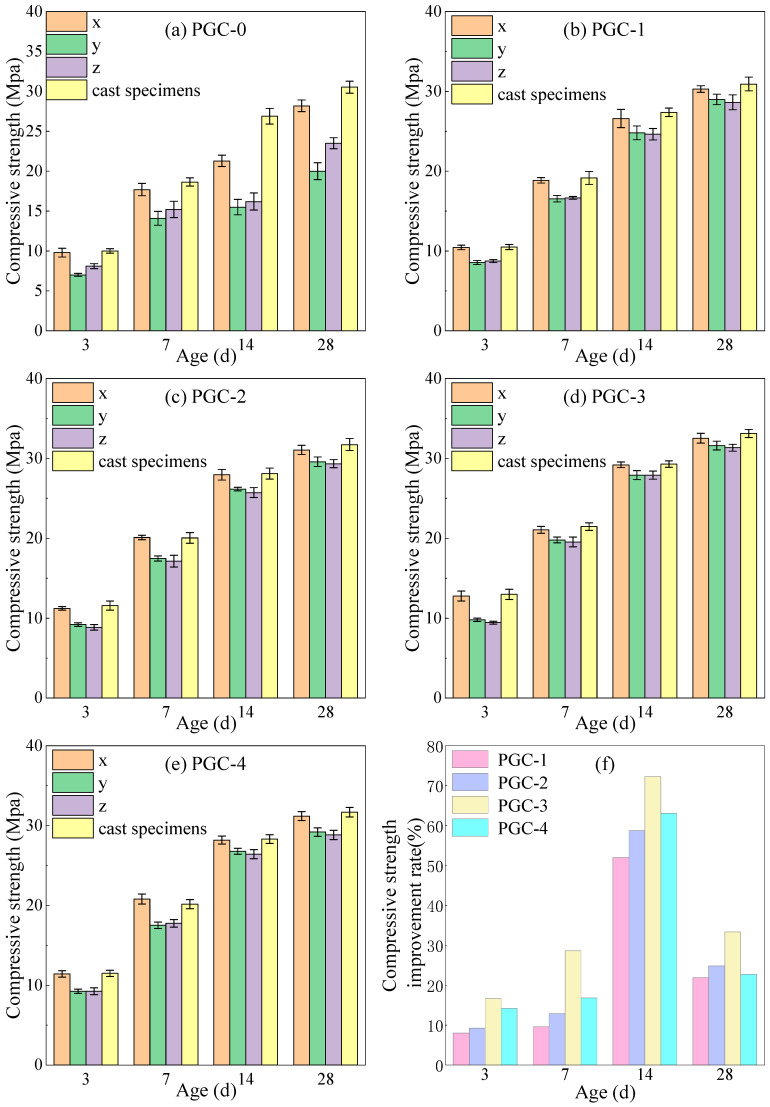
Compressive strength results (mean ± SD, n = 3). (**a**) Control group. (**b**) CRR is 50%. (**c**) CRR is 60%. (**d**) CRR is 70%. (**e**) CRR is 80%. (**f**) Compressive strength increase (%) over PGC-0.

The compressive-strength test results for specimens from PGC-1 to PGC-4 and PGC-0 at the ages of 3 d, 7 d, 14 d, and 28 d are presented in Figure 15. Consistent with the trend observed for flexural strength, the influence of the CRR on the 3D-printed PGC compressive-strength culminates at a CRR of 70%, at which point the maximum value is attained.

According to Figure 15a–e, upon partial cement replacement by BS, FA, and SF, the 3DPPGC exhibited a consistent enhancement in compressive strength across all curing ages. Analogous to the flexural-strength response, the compressive strength of 3DPPGC first increased and then decreased with the rising CRR; nevertheless, all age-dependent strengths remained below those of the cast counterparts. At a CRR of 70%, the maximum compressive strengths in the x-direction reached 98.35%, 98.14%, 99.66%, and 98.19% of the cast values at 3 d, 7 d, 14 d, and 28 d, respectively. Conversely, along the z-direction, which is the actual principal load-bearing orientation, the corresponding strengths only ranged from 72.73% to 95.25% of the cast values across the same curing ages. As shown in Figure 15f, all groups from PGC-1 to PGC-4 had higher z-direction compressive strength than PGC-0 at all ages. PGC-3 performed the best, with increases of 16.63%, 28.63%, 72.24%, and 33.35% at 3, 7, 14, and 28 days, respectively. Overall, the strength improvement rate increased with age, peaked at 14 days, and reached a maximum of 72.24%.

Table 6 presents the mean rate of increase in compressive strength in the x-direction for the PGC-0–PGC-4 series and the cast specimens.

As demonstrated by the data presented in Table 6, the incorporation of BS, FA, and SF markedly accelerates the early-age compressive-strength development of 3DPPGC, particularly within the first three days. When the CRR reaches 70%, the compressive-strength growth rates of 3DPPGC specimens during 0–3 d, 7–14 d, and 14–28 d all exceed those of the other groups; even at 3–7 d, the growth rate deviates from the maximum by merely 0.5. These observations demonstrate that 3DPPGC with a CRR of 70% achieves the optimal compressive-strength development kinetics.

**Figure 16:**
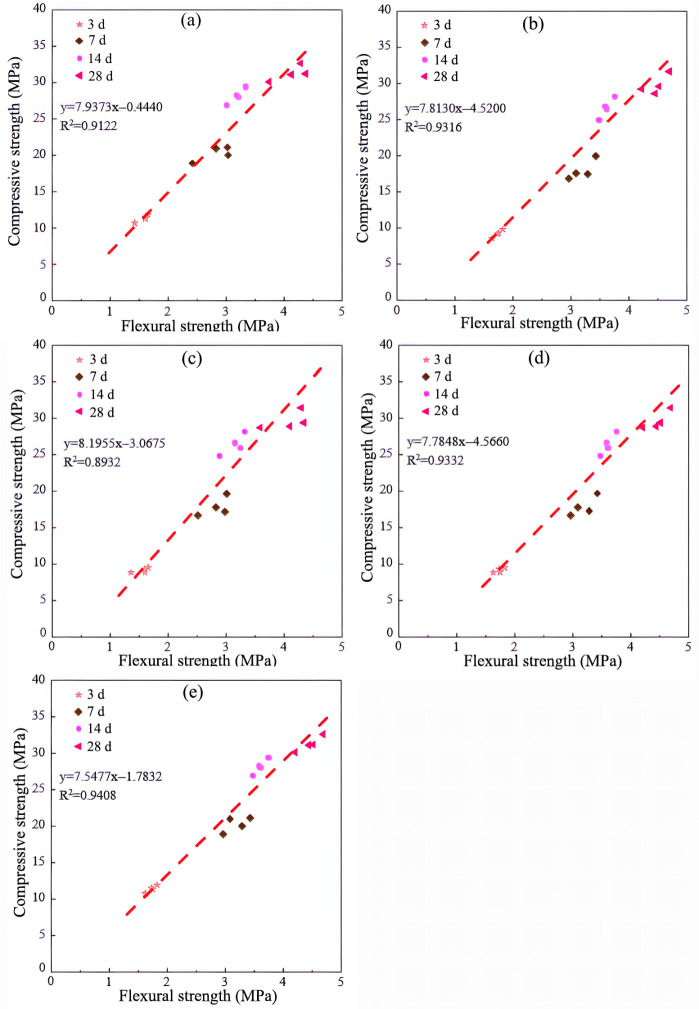
Relationship between flexural and compressive strengths of 3DPPGC with different CRRs at various curing ages under distinct loading directions. (**a**) x-direction. (**b**) y-direction. (**c**) z-direction. (**d**) Flexural strength in the y-direction versus compressive strength in the z-direction. (**e**) Flexural strength in the y-direction versus compressive strength in the x-direction.

In general, the flexural-strength of concrete exhibits an approximately linear correlation with its compressive strength. As illustrated in Figure 16a–c, the results of linear regression between the compressive strength and the flexural strength of the 3D-printed PGC with various CRRs in the x, y, and z directions yielded coefficients of R^2^ of 0.9122, 0.9316, and 0.8932, respectively, indicating a pronounced linear relationship. The deviation from one is attributable to experimental errors inherent in the flexural and compressive tests.

In practical engineering applications, the flexural strength is measured along the y-direction depicted in Figure 8a, whereas the compressive strength is evaluated along the z-direction shown in Figure 8b. As illustrated in Figure 16d, these two quantities exhibit a strong linear correlation with an R^2^ value of 0.9332. Figure 16e presents the maximum flexural and compressive strengths of each 3DPPGC group at different curing ages, with the flexural strength again determined along the y-direction in Figure 8a and the compressive strength along the x-direction in Figure 8b. The two strength parameters likewise display a linear relationship, characterized by an R^2^ value of 0.9408.

#### 4.5.3. Strengthening Mechanism of BS and PG in 3DPPGC

The mechanism for strength improvement in PG composite cementitious concrete can be summarized as follows: In an alkaline environment, BS releases Ca^2+^, which triggers a chain reaction. The Ca^2+^ undergoes an ion exchange reaction with cancrinite, releasing Na^+^ [54]. These sodium ions usually exist in the form of NaOH, thereby increasing the alkalinity of the system. This further activates the silicon and aluminum components in the cement and BS, promoting their reaction with the hydration product calcium hydroxide (CH) to form gel phases such as amorphous calcium silicate hydrate (C-S-H) and calcium aluminate hydrate (C-A-H), while simultaneously consuming CH crystals. Research indicates that variations in CH content and C-S-H yield can effectively characterize the evolution of hydration-product strength; a reduction in CH is generally accompanied by strength enhancement [55,56]. This process further accelerates the hydration of dicalcium silicate (C_2_S) and tricalcium aluminate (C_3_A) [57], generating calcium–aluminosilicate–hydrate (C-(A)-S-H), which is the principal mechanism underlying strength development.

The incorporation of PG and the sulfate ions (SO_4_^2−^) it supplies play a pivotal role in enhancing the performance of 3DPPGC [58,59,60]. Prior investigations have demonstrated that SO_4_^2−^ markedly accelerates ettringite formation, with its precipitation rate exhibiting a direct, positive correlation with sulfate concentration, while simultaneously altering the charge distribution within the system [54]. Furthermore, SO_4_^2−^ can promote the dissolution of Si^4+^ and Al^3+^ in cement and BS, increase the formation of C-(A)-S-H gels, and change the water permeability of C-S-H by solid solution to C-S-H or adsorption, and accelerate the formation of C-S-H [61]. This synergistic effect significantly improves the strength of the composite cementitious material. In the context of the macroscopic behavior observed in this study, specifically the continuous strength-enhancement as the CRR increased to 70%, it can be inferred that a synergistic activation effect was successfully achieved. The incorporation of BS evidently increased the content of reactive aluminum and silicon available for hydration. It is hypothesized that the effective supply of SO_4_^2−^ from the 20% PG dosage acted as a chemical activator, which not only accelerated the reaction of pozzolanic materials [58,62,63] but also altered the charge distribution [57] and optimized the C-S-H structure [54]. This correlates well with the rapid early-age strength development observed in the PGC-3 (70% CRR) specimens, suggesting that the balance between alkalinity provided by the cement/lime and the reactivity of the BS/PG was optimal at this ratio. However, regarding the strength decline observed when the CRR exceeded 70% (PGC-4), the mechanism can be attributed to the “alkalinity dilution” effect. While BS contributes reactive phases, its activation is heavily dependent on the alkalinity of the system [64]. We hypothesize that once the BS dosage exceeded the critical threshold relative to the Portland cement content, the total alkalinity of the system became insufficient to fully break the Si-O and Al-O bonds in the slag. Consequently, despite the higher potential for C-S-H formation, the reaction rate decelerated, leading to the observed decrease in both flexural and compressive strengths at 80% CRR. This confirms that optimizing the BS dosage is essential to maintain the delicate balance between providing sufficient reactive precursors and sustaining the necessary alkaline environment for hydration. The efficient supply of SO_4_^2−^ not only accelerates the reaction of volcanic ash materials [58,62,63], but also provides an effective path for the realization of high-strength 3DPC, including promoting the formation of calcare, adjusting the charge distribution, and optimizing the C-S-H structure.

**Figure 17:**
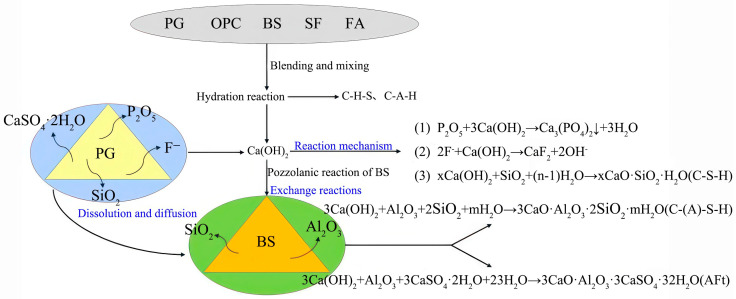
Strengthening mechanism of PGC.

The strength-enhancement mechanism of PGC is shown in Figure 17. Studies have shown [65] that Ca (OH)_2_ can react with SiO_2_ in PG, and BS, as an important source of active SiO_2_ and Al_2_O_3_, provides the necessary aluminum phase and siloxane groups for the formation of calcarelite and C-(A)-S-H gelitols [52]. The activation of BS can only be fully exerted in an alkaline- and sulfate-containing environment [64]. In an alkaline environment, CaO and Al_2_O_3_ in BS react with OH^−^ to release Ca^2+^ and Al^3+^, and sulfate promotes the formation of calcium sulfaluminate hydrate with SO_4_^2−^, reducing the concentrations of Ca^2+^ and Al^3+^ in solution [66], breaking the equilibrium state, thereby accelerating the dissolution of CaO and Al_2_O_3_ in BS and stimulating their hydration activity [52]. In addition, SO_4_^2−^ can also promote the dissolution of Al^3+^ and Si^4+^ in BS, further increasing the formation of cementitious materials and ultimately improving the mechanical properties of PGC.

Table 7 compares this research with the previous literature on PG-based materials. Unlike Tarhan et al. [37], whose work used only a low phosphogypsum content (5–7.5%), this study achieves up to 70% cement replacement (with a PG mass fraction of 20%) while ensuring excellent printability and maintaining mechanical strength that meets structural requirements. Furthermore, unlike Sinka et al. [32], who used calcined PG to produce low-strength materials, this study proposes a processing route without calcination. It directly utilizes the sulfate in raw PG to activate slag reactivity. This method not only simplifies the pretreatment process and reduces energy consumption but also effectively addresses the common challenge of significant strength-reduction with high solid-waste content. It achieves a balance between high solid-waste utilization and high performance. However, the main limitation of this multi-solid-waste system is its sensitivity to fluctuations in the chemical composition of raw materials (such as impurity content in PG), which requires strict quality control in future large-scale applications.

## 5. Conclusions

(1) This study systematically investigates the influence of the CRR on the PGC. Experimental results demonstrate that the incorporation of BS markedly accelerates the early-age hydration kinetics of PGC; the setting time reaches its minimum when CRR is 70%. Flow-table tests indicate a pronounced loss of flowability within 10 min, attributable to the accelerated hydration. Extrudability assessments reveal that all PGC-1 to PGC-4 mix designs exhibit excellent extrusion performance. With respect to buildability, the 3DPPGC specimen prepared at a CRR of 70% achieves the highest performance, yielding a J_z_ value of 99.53%, thereby fully satisfying the buildability requirements for 3D printing.

(2) The influence of incorporating BS, FA, and SF on the strength evolution of 3DPPGC was systematically investigated. The joint addition of BS, FA, and SF enhanced the strength of 3DPPGC across all curing ages. When the CRR was set at 70%, the early-age strength of 3DPPGC was markedly elevated, and the later-age compressive strength was likewise significantly improved. Specifically, the 28-day flexural strength of 3DPPGC specimens printed in the y-direction surpassed that of conventionally cast counterparts by 1.52%. Furthermore, the incorporation of BS, FA, and SF substantially accelerated the early-age strength development rate of the material.

(3) This study advances the theoretical framework for solid-waste utilization in additive manufacturing by demonstrating that uncalcined PG can act as an effective in situ sulfate activator for slag-based 3D-printing paste. A critical “alkali–sulfate balance” mechanism is elucidated: at a 70% CRR, the synergistic formation of ettringite and C-S-H gel maximizes both the early-age buildability and the long-term strength of the material, resulting in optimal mechanical performance—a 1.52% enhancement over conventional cast concrete. Conversely, exceeding this CRR induces an “alkalinity dilution” effect, which impedes microstructure formation. These findings establish a theoretical foundation for designing low-carbon, multi-solid-waste cementitious composites tailored for smart construction.

## Figures and Tables

**Table 1:** Chemical composition of the cementitious materials employed in this study (wt%).

Constituents	PG	OPC	SF	BS	FA
CaO	36.212	59.122	0.512	41.213	2.720
SiO_2_	7.032	22.654	92.511	35.153	57.26
SO_3_	39.158	2.715	2.111	1.159	0.125
Al_2_O_3_	1.108	5.266	0.082	13.556	30.562
Fe_2_O_3_	0.568	3.325	0.092	0	1.156
MgO	0.125	2.215	0.633	3.561	0.685
K_2_O	0.806	0.812	0.531	0.259	1.562
TiO_2_	0.123	0.516	0.297	0.112	2.756
H_2_O	10.445	0	0	0	0
Others	4.423	3.375	3.231	4.987	3.174
Sum	100	100	100	100	100

**Table 2:** Mix proportions of the cementitious materials.

No.			Mass/g		
	PG	OPC	BS	FA	SF
0	1200	4800	0	0	0
1	1200	3000	1170	420	210
2	1200	2400	1896	336	168
3	1200	1800	2622	252	126
4	1200	1200	3348	168	84

**Table 3:** Mix proportions of PGC at varying CRRs.

No.						Mass/g					
	CRR	PG	OPC	BS	FA	SF	Sand	CaO	CL	WR	TEOA
PGC-0	20%	1200	4800	0	0	0	6000	96	20	10	1
PGC-1	50%	1200	3000	1170	420	210	6000	96	20	10	1
PGC-2	60%	1200	2400	1896	336	168	6000	96	20	10	1
PGC-3	70%	1200	1800	2622	252	126	6000	96	20	10	1
PGC-4	80%	1200	1200	3348	168	84	6000	96	20	10	1

**Table 4:** Printing parameters.

Layer Height (mm)	Line Width (mm)	Infill Density (%)	Print Speed (mm/s)	Extrusion Flow Rate (cm^3^/s)
12.5	25	100	50	2.3

**Table 5:** Growth rate of the flexural strength in the y-direction for 3DPPGC with varying CRRs.

CRR	Average Rate of Increase in Flexural Strength (×10^−2^ MPa/d)			
	0–3 d	3–7 d	7–14 d	14–28 d
Cast specimen	60.70	38.64	6.37	5.78
20%	48.67	16.25	10.71	7.07
50%	54.33	33.5	7.34	5.21
60%	58.33	38.50	6.43	4.57
70%	60.67	40.25	6.71	4.59
80%	57.67	34.00	6.07	4.27

**Table 6:** Growth rate of the compressive strength in the x-direction for 3DPPGC with varying CRRs.

CRR	Average Rate of Increase in Compressive Strength (×10^−1^ MPa/d)			
	0–3 d	3–7 d	7–14 d	14–28 d
Cast specimen	40.33	23.38	11.56	2.62
20%	33.33	22.49	8.25	4.87
50%	35.67	20.50	11.43	2.29
60%	37.67	21.75	11.43	2.29
70%	39.67	23.00	11.86	2.29
80%	38.33	23.50	10.43	2.07

**Table 7:** Comparison of this study with previous research on PG-based cementitious materials.

	Forming Method	Mix Proportion (wt.%)	Compressive Strength	Flexural Strength
Huang et al. [40]	casting	PG: 45% Steel slag (SS): 10% Ground granulated blast furnace slag (GGBFS): 35% Limestone (LS): 10%	28-day compressive strength greater than 40 MPa.	28-day flexural strength greater than 8 MPa.
Liu et al. [36]	casting	PG: 45% Ground Granulated Blast Furnace Slag (GGBFS): 53% Lime: 2%, Sodium Aluminate: 1 wt.%.	The 28-day compressive strength is 18.25 MPa. The 3 d compressive strength of specimens after adding 0.2 wt.%, 0.5 wt.%, 0.8 wt.%, and 1 wt.% sodium aluminate increased by 82.26%, 309.19%, 491.88%, and 587.39%, respectively.	
Sahmenko et al. [35]	3D printing	Recycled Gypsum (RG), PG: 55% Metakaolin (MK): 22.5% Portland Cement (CEM I): 22.5%	Gypsum–cement–pozzolanic (PG-based): 28-day compressive strength, wet = 29.6 MPa, dry = 38.6 MPa.	
Tarhan et al. [37]	3D printing	Cement: 60%, FA or ground granulated blast-furnace slag (GGBS) as supplementary cementitious materials account for 20%; Gypsum accounts for 20%, in which PG and borogypsum serve as partial replacements for gypsum (replacement ratio ranges from 2.5% to 10%).	With the addition of 7.5% PG to the GGBS system, the 28-day compressive strength reached 51 MPa.	
Sinka et al. [32]	3D printing	40% binder (consisting of calcined PG and 0.5% quicklime) and 60% sand.	Printed specimen (u-direction): 7-day compressive strength 0.95 MPa Cast specimen: 7-day compressive strength 0.81 MPa	Printed specimen: 7-day tensile strength 0.83 MPa, 0.92 MPa Cast specimen: 7-day tensile strength 0.71 MPa
This study	3D printing	PG: 20%; Using PG, blast furnace slag (BFS), FA (FA), and SF as replacements for cement (CRR = 50–80%).	CRR = 70%: the 28-day printed specimen (in the x-direction) achieves 98.19% of the compressive strength of cast specimens, while the compressive strength in the z-direction ranges from 72% to 95% of that of cast specimens.	CRR = 70%: The 28-day printed sample (Y-direction) achieved 4.69 MPa, which is 1.52% higher than the cast sample.

## Data Availability

The original contributions presented in this study are included in the article. Further inquiries can be directed to the corresponding author.

## Abbreviation

The following abbreviations are used in this manuscript:

3DPPGC	3D-printed phosphogypsum concrete
3DPC	3D-printed concrete
PG	Phosphogypsum
GCP	Gypsum–cement–pozzolan
CRR	Cement replacement ratio
BS	Blast-furnace slag
FA	Fly ash
SF	Silica fume
PGC	Phosphogypsum concrete
WR	Polycarboxylate-based high-range water-reducing admixture
TEOA	Triethanolamine
